# Estradiol Increases Microglial Response to Lipopolysaccharide in the Ventromedial Hypothalamus during the Peripubertal Sensitive Period in Female Mice

**DOI:** 10.1523/ENEURO.0505-19.2020

**Published:** 2020-07-02

**Authors:** Amarylis Velez-Perez, Mary K. Holder, Sam Fountain, Jeffrey D. Blaustein

**Affiliations:** 1 Department of Psychological and Brain Sciences, University of Massachusetts, Amherst, MA 01003; 2 Neuroscience and Behavior Program, University of Massachusetts, Amherst, MA 01003; 3 Center for Neuroendocrine Studies, University of Massachusetts, Amherst, MA 01003

**Keywords:** estradiol, Iba1, mice, microglia, peripubertal development

## Abstract

Sensitive periods are times of development during which the effects of experience are unusually strong and long lasting. The peripubertal period has emerged as one such sensitive period, and a single administration of lipopolysaccharide (LPS) during this time reduces hormone-induced sexual behavior in adult female mice. During periods of high synaptic turnover, maturation, and elimination, as occurs during this sensitive period, microglia are particularly active. Estradiol also regulates microglial numbers, morphology, and activation. In addition, a good deal of evidence suggests that estradiol may confer this vulnerability to the effects of a stressor during the peripubertal period. Therefore, we investigated the effects of estradiol on microglial morphology, cytokine levels, and the sickness response to LPS. Estradiol levels were manipulated by implanting an estradiol-filled SILASTIC capsule (or oil-filled control) in ovariectomized mice or by administering the aromatase inhibitor, formestane (or oil control), to ovary-intact mice. We found that (1) estradiol elevates basal microglial Iba1 immunoreactivity in the ventromedial nucleus of the hypothalamus (VMH), (2) LPS induces higher levels of proinflammatory cytokines in the presence of estradiol, and (3) LPS causes hypothermia in the presence of estradiol. Taken together, these data suggest that estradiol enhances the effect of LPS during the pubertal sensitive period.

## Significance Statement

The experience of trauma or extreme stressors, including immunostressors, during the peripubertal period can lead to marked and pervasive alterations of behavior. The underlying mechanisms contributing to this vulnerability during the peripubertal period are not understood. Microglia have emerged as a candidate through which stress might impact neurodevelopment and subsequently behaviors. Few studies have investigated how the developmental changes in levels of estrogens during the peripubertal period influence the microglia. Here, we find that exposure to estradiol during the peripubertal period enhances the microglial response to an immune challenge. These findings suggest a mechanism through which estradiol may confer vulnerability to an immunostressor during the sensitive, peripubertal period.

## Introduction

Sensitive periods are developmental windows during which the effects of experience can lead to enduring effects (Knudsen, 2004). Stressful experiences can perturb normal development processes to reorganize brain circuits regulating behavior (Avital and Richter-Levin, 2005; Blaustein and Ismail, 2013; Green and McCormick, 2013). One such sensitive period is pubertal development as indicated by a comprehensive series of experiments on the age vulnerability on the behavioral sequelae (Laroche et al., 2009b). In particular, an immune challenge like the bacterial endotoxin, lipopolysaccharide (LPS), or stress from shipping during pubertal development, but not other times, decreases subsequent hormone-induced female sexual behavior in adult female mice (Ismail et al., 2011; Olesen et al., 2011; Laroche et al., 2009a,b). While males shipped at six weeks also show reduced mounts in a sexual behavior test, this effect is less robust than in females (Laroche et al., 2009b); therefore, we have chosen to focus on effects of peripubertal LPS in females.

During sensitive periods of development, there are marked changes in the architecture of brain regions via cell genesis, cell death, and synaptic remodeling (Tremblay et al., 2010; Schafer et al., 2012). Moreover, microglia, the resident-immune cells of the brain, are particularly active during these processes (Paolicelli and Gross, 2011), suggesting that the role of microglia in monitoring the brain microenvironment is particularly important during sensitive periods of development. For example, the complement system, part of the innate immune system, instructs microglia to engulf and eliminate immature synapses (Stevens et al., 2007). Disruption of key signaling molecules in this pathway alters the establishment of neural circuits (Hong and Stevens, 2016). One of the signaling molecules secreted by microglia that is crucial for normal developmental processes are cytokines (Bilbo and Schwarz, 2009). Therefore, it is likely that an inflammatory insult like an LPS injection can alter the cytokine signaling, permanently remodeling neural circuits (Bilbo and Schwarz, 2012). In fact, a bacterial infection or LPS administration to neonatal male rats increases brain cytokines levels, inducing long-term changes in microglial activation and behavior (Bilbo et al., 2005, 2008). Juvenile and adult female rats, however, have a more activated phenotype of microglia, compared with males (Schwarz et al., 2012). Thus, microglia may mediate the enduring effects of LPS in pubertal female mice, and indeed, a single injection of LPS causes a stronger microglial activation in peripubertal mice compared with adult mice receiving the same LPS treatment (Holder and Blaustein, 2017). Overall, these data suggest that microglia are particularly active during sensitive periods of development, and further activation by an immune challenge may have implications for the establishment of neuronal circuits regulating behavior.

One of the hallmarks of the peripubertal period is the maturation of the reproductive axis, resulting in the secretion of gonadal steroid hormones. As these gonadal hormones impact neurobiological processes regulating reproduction at the molecular, cellular, physiological, and behavioral levels, it is likely that estradiol may shape these neuronal circuits during pubertal development. For example, full expression of female sexual receptivity in adulthood depends on peripubertal secretion of estradiol (Bakker et al., 2003). Aromatase knockout females display low levels of hormone-induced sexual behavior in adulthood (Bakker et al., 2003), and exposure to peripubertal, but not prepubertal (post-natal day; P5−P15), estradiol rescues adult sexual behavior (Brock et al., 2011). This suggests that the timing and exposure of the brain to gonadal hormones are critical factors driving the full maturation of the reproductive behavioral repertoire.

One way in which estradiol can shape neuronal circuits is by regulating microglia. Microglia express estrogen receptors (ER; Sierra et al., 2008), suggesting that estrogens can modulate the onset, duration, and/or culmination of neuroinflammation. In addition, estradiol can regulate microglial numbers, morphology and cytokine expression at basal levels (Mor et al., 1999) and after an immune challenge (Vegeto et al., 2001). Therefore, we hypothesized that estradiol confers vulnerability to LPS by enhancing the immune response during the peripubertal development in female mice. To test this hypothesis, we manipulated estradiol levels by administering an estradiol capsule to ovariectomized mice or by administering the aromatase inhibitor formestane (or oil to the control group). We quantified changes in the morphometric properties of microglia in the ventromedial nucleus of the hypothalamus (VMH), we examined the effects of LPS on interleukin (IL)-1α, IL-1β, IL-6, IL-10, and tumor necrosis factor (TNF)-α protein levels, and we quantified changes in behavior (lethargy), physiology (thermoregulation), and metabolism (body weight), all of which are critical components of sickness behavior and constitute an organized response to immune activation (Dantzer, 2001).

## Materials and Methods

### Animals

Female C57Bl/6 mice were shipped from Charles River Laboratories on P21 and housed in an all-female colony room. The room was under controlled temperature (24 ± 2°C) and reversed 14/10 h light/dark cycle (zeitgeber time was 8 P.M., with lights off at 10 A.M.). Mice were housed in groups of four in clear polycarbonate cages with *ad libitum* access to food (Teklad 2014, phytoestrogen-reduced diet, Harlan Laboratories) and water in glass bottles. All procedures were performed according to the National Institutes of Health Guide for the Care and Use of Laboratory Animals and in accordance with the University of Massachusetts, Amherst Institutional Animal Care and Use Committee’ regulation. Experimenters were blinded to all experimental treatments.

### Ovariectomy and capsule implantation

On P28, mice were anesthetized with isoflurane (3%, inhaled), and a single incision was made to the dorsal skin and the muscle layer to locate the uterine horns. The uterine horns were clamped, the ovaries were removed, and the tips of the uterine horns were cauterized with an Acu-Cautery (Acuderm Inc). The muscle layer was sutured with an absorbable suture (Ethicon), and the incision was closed using surgical wound clips (Becton Dickinson). For capsule implantation, a small incision was made to the skin layer of the neck, and a 2.5-cm-long SILASTIC capsule (1.57 mm inner diameter × 3.18 mm outer diameter; Dow Corning) filled with estradiol or sesame oil was implanted (Knox et al., 2009). The incision was closed using surgical wound clips. Mice were transferred to a heating pad to recover until they were fully awake, and they were returned to their home cage.

### E-mitter implant

During the ovariectomy procedure, mice received a G2 E-mitter device (E-mitter Respironics Mini-mitter) to record core body temperature and activity levels. The E-mitter was sutured to the abdominal muscle wall to keep its placement stable. Before implantation, the E-mitter was washed with Tergazyme, a detergent with protease enzyme activity. Following Tergazyme, E-mitters were incubated in 3% glutaraldehyde to disinfect and sterilize the E-mitter and rinsed with sterile saline at least three times. Radio signals for locomotion and temperature were recorded by a receiver board (ER-4000 energizer receiver) underneath the cage housing each animal and stored via Vital View Data Acquisition System (version 4.1; Mini Mitter) in the laboratory computer. Mice were allowed to recover from E-mitter implant surgery for at least one week before onset of the experiments. Data were collected in 2-min bins, starting 24 h before the injection until decapitation (at either 6 or 24 h following LPS or vehicle injection).

### Estradiol administration

United States Pharmacopeia (USP) grade 17β-estradiol E_2_ was obtained from Sigma-Aldrich. Estradiol was dissolved in sesame oil (vehicle) to a concentration of 50 μg/25 μl. Mice were randomly assigned to treatment groups and received a SILASTIC capsule (Dow Corning) filled with estradiol (50 μg E_2_/25 μl sesame oil) or sesame oil.

### Aromatase inhibitor formestane

Biosynthesis of estrogens is catalyzed by the rate-limiting, cytochrome P450 enzyme, aromatase (Yue and Brodie, 1997). This can be inhibited by aromatase inhibitors like formestane (4-hydroxyandrostendione), which acts as a steroidal substrate analog (Yue and Brodie, 1997; Simpson and Dowsett, 2002). Mice were subcutaneously injected with formestane or sesame oil for seven consecutive days, starting on P35 through P42 at a dose of 20 μg/kg. This dose of formestane decreases neural estradiol levels *in vivo* in neonatal rats (Amateau et al., 2004). In addition, this dose and injection schedule reduces estradiol-dependent changes in dendritic spine morphology in P7–P13 perinatal rats (Dean et al., 2012).

### LPS treatment

LPS from *Escherichia coli* serotype 026:B6 was obtained from Sigma-Aldrich. The LPS was dissolved in sterile saline (vehicle) to a concentration of 0.1 mg/ml. Mice were randomly assigned to treatment groups and received a single intraperitoneal injection of LPS (1.5 mg/kg body weight) or an equivalent volume of sterile saline on P42. Injections were administered within 1 h before the onset of the dark phase of the light/dark cycle. Animals were returned to their home cage immediately following injection. Mice were weighed immediately before injection, as well as 6 and 24 h following treatment to assess the effect of LPS on body weight.

### Tissue collection

Mice were deeply anesthetized with an intraperitoneal injection of pentobarbital (100 mg/kg) and decapitated. Brains were removed from the skull and fixed in 4% paraformaldehyde in PBS (0.05 m, pH 7.4) at 4°C for 24 h, followed by incubation in 30% sucrose in 0.1 m PBS for at least 72 h. Brains were sectioned coronally in 30-μm-thick sections using a cryostat. Brain sections were stored in cryoprotectant at −20°C until they were immunostained.

### Iba1 immunocytochemistry

Free-floating brain sections were rinsed for 3 × 5 min in 0.05 m tris-buffered saline (TBS) and also rinsed before each subsequent step, except between the blocking and primary antibody steps. Brain sections were incubated in a blocking buffer containing 20% normal goat serum, 3.5% hydrogen peroxide, and 1% bovine serum albumin in gel TBS for 1 h. Brain slices were incubated for 48 h at 4°C in a solution containing Iba1 primary antibody (Wako catalog #019-19 741, RRID:AB_839504) at a 1:10,000 dilution. The primary Iba1 antibody was diluted in 2% normal goat serum and 0.5% Triton X-100 in gel TBS. After 48 h, sections were incubated for 90 min at room temperature in a solution of biotinylated secondary antibody in gel TBS. The avidin-biotin complex (ABC) was used to bind a complex of streptavidin-biotin peroxidase to the secondary antibody for 90 min. The reaction was then developed with diaminobenzidine (DAB; Vector Laboratories) for 20 min to produce a colorimetric stain. Brain sections were rinsed in TBS and mounted on gelatin-coated slides, dehydrated, and coverslipped with Polymount.

### Microscopic analysis of Iba1

Three bilateral photomicrographs of carefully matched sections of VMH were taken from each animal using a Nikon Optiphot-2 microscope connected to a QImaging Micropublisher RTV 5.0 camera. The identification of this area was based on plate 43–44 of the *Mouse Brain Atlas in Stereotaxic Coordinates*, using the shape of the third ventricle as a landmark (Franklin and Paxinos, 1997). Gray-scale images of the VMH were obtained using the ImageJ software (NIH Image, Scion Image), and Iba1 staining was measured using a maximum entropy thresholding algorithm approach, a widely used broad approach to measure Iba1 immunoreactivity (Beynon and Walker, 2012). Detailed morphometric analysis of individual microglia was obtained using the MetaMorph Neurite Outgrowth program (Molecular Devices; version 7.7). We measured changes in microglia morphology by quantifying the number of microglia, total cell body size, mean cell body area, total outgrowth, mean outgrowth, total branches, and total processes. While the quantification of global changes in Iba1 immunoreactivity is common, it can lead to the incorrect assumption that microglia have the same function or that their effects are additive, because the data are reported as an average profile (Beynon and Walker, 2012; Gertig and Hanisch, 2014). In order to capture microglial heterogeneity and morphologic changes after LPS within the VMH, we performed a ratio analysis that involved quantifying microglial cell body size over microglial ramifications (Hovens et al., 2015). A ratio between 0.00 and 0.99 reflects properties of highly ramified microglia (basal and/or surveying microglia), whereas a ratio of 3.00 and above reflects activated microglia (large somata and short or nearly non-existent processes, associated with a phagocytic stage). Microglia with stout processes (intermediate ratio 1.0–2.99) can function as dendritic cells (Hanisch, 2013). As mice were not perfused before collecting the brains, it is possible that the Iba1 staining may reflect microglia but also monocytes and macrophages near the blood vessels. Brain regions were identified using the *Mouse Brain Atlas in Stereotaxic Coordinates*, plates 43–44 (Franklin and Paxinos, 1997).

### Plasma

Mice were deeply anesthetized with an intraperitoneal injection of pentobarbital (100 mg/kg) and decapitated. Trunk blood was collected into an EDTA-treated tube (BD Vacutainer) and placed on ice. Blood was centrifuged at 4°C in a microcentrifuge (Eppendorf). Following centrifugation, plasma was aliquoted and stored at −80°C until assayed. Estradiol levels were determined using the mouse estradiol ELISA kit (Calbiotech). The level of detection is 3 pg/ml, which is consistent with the results of McNamara et al. (2010), in which the levels of estradiol were found to be <5 pg/ml in adult female mice.

### Cytokine protein quantification

Cytokine protein levels in plasma were measured 6 and 24 h after LPS administration. The 6-h time point was selected because, in a time course profile of cytokine levels 6 h post-LPS, this was the optimal time point to measure the peak levels of cytokines of interest. It is also the time point at which a single peripheral LPS injection causes significant breakdown of the blood brain barrier (Brown et al., 2010). The 24-h time point was selected to determine how cytokine levels at this time point relate to microglial changes. The levels of IL-1α, IL-1β, IL-6, IL-10, and TNF-α were analyzed using the Luminex Multiplex Instrument Bio-Plex 200 system from samples shipped to the Mouse Metabolic and Phenotyping Center at the University of Massachusetts Medical School.

### Statistics

Data are represented as means ± SEM. We examined the effect of treatment (saline and LPS) and hormonal milieu (estradiol or oil capsule in ovariectomized mice or formestane and oil controls in ovary-intact mice) on Iba1 immunoreactivity, cytokine protein levels, body weight, and estradiol levels using a two-way ANOVA. We corrected for multiple comparisons using Bonferroni corrections. All data in experimental 1 were normally distributed except for the VMH mean cell body area, the VMH total outgrowth, the VMH total branches, and the IL-1α 24 h. All data in experiment 2 were normally distributed except for VMH number of microglia, the total cell body area, the mean cell body area, IL-10 6 h, IL-1α 24 h, IL-1β 24 h, IL-10 24 h, TNF-α 24 h, the changes in body weight at 6 and 24 h, and the change in temperature. The statistical tests and observed powers were conducted using GraphPad Prism. Effect sizes estimates were calculated using η^2^ for the ANOVAs (Levine and Hullett, 2002). Each comparison is indicated using superscript letters and numbers are listed in Tables 1, 2.

**Table 1:** Experiment 1 statistical analysis

	Dependent variable	Comparisons	*p* value	Effect size	Power
a^1^	VMH mean Iba1-ir	Main effect of hormones Main effect of treatment Interaction	0.0047 <0.0001	0.350 0.747	0.207
a^2^	VMH number of microglia	Main effect of hormones Main effect of treatment Interaction	0.0261 <0.0001	0.234 0.822	0.207
a^3^	VMH total cell body area	Main effect of hormones Main effect of treatment Interaction	0.0250 <0.0001	0.238 0.926	0.207
a^4^	VMH mean cell body area	Main effect of hormones Main effect of treatment Interaction	0.1917 <0.0001	0.088 0.901	0.207
a^5^	VMH total outgrowth	Main effect of hormones Main effect of treatment Interaction	0.895 <0.0001	0.001 0.718	0.207
a^6^	VMH mean outgrowth	Main effect of hormones Main effect of treatment Interaction	0.2159 <0.0001	0.079 0.866	0.207
a^7^	VMH total processes	Main effect of hormones Main effect of treatment Interaction	0.1779 <0.0001	0.094 0.713	0.207
a^8^	VMH total branches	Main effect of hormones Main effect of treatment Interaction	0.6455 <0.0001	0.011 0.773	0.207
a^9^	VMH microglial ratio	Main effect of ratio Main effect of treatment Interaction	<0.0001 0.04 <0.0001	0.424 0.103 0.742	0.207
b^1^	IL-1α 6 h	Main effect of hormones Main effect of treatment Interaction	0.495 0.781 0.077	0.043 0.011 0.256	0.144
b^2^	IL-1β 6 h	--	--	--	--
b^3^	IL-6 6 h	Main effect of hormones Main effect of treatment Interaction	0.667 0.008 0.664	0.019 0.517 0.019	0.136
b^4^	IL-10 6 h	Main effect of hormones Main effect of treatment Interaction	0.008 <0.0001 0.008	0.482 0.769 0.492	0.144
b^5^	TNF-α 6 h	Main effect of hormones Main effect of treatment Interaction	0.053 <0.0001 0.017	0.132 0.938 0.451	0.136
b^6^	Change in body weight 6 h	Effect of hormone Main effect of treatment Interaction	0.2662 <0.0001 0.0258	0.065 0.902 0.235	0.207
c^1^	IL-1α 24 h	Main effect of hormones Main effect of treatment Interaction	0.333 0.0003 0.031	0.059 0.562 0.260	0.183
c^2^	IL-1β 24 h	Main effect of hormones Main effect of treatment Interaction	0.718 0.514 0.379	0.049 0.153 0.261	0.077
c^3^	IL-6 24 h	Main effect of hormones Main effect of treatment Interaction	0.631 0.0003 0.117	0.101 0.623 0.165	0.167
c^4^	IL-10 24 h	Main effect of hormones Main effect of treatment Interaction	0.2599 0.085 0.26205	0.096 0.211 0.019	0.159
c^5^	TNF-α 24 h	Main effect of hormones Main effect of treatment Interaction	0.257 0.739 0.6751	0.019 0.054 0.014	0.159
c^6^	Change in body weight 24 h	Effect of hormone Main effect of treatment Interaction	0.792 <0.0001 0.099	0.003 0.816 0.129	0.207
d^1^	Estradiol	Main effect of hormone	<0.0001	0.840	0.269
e^1^	Change in temperature	Main effect of time Main effect of treatment Interaction	<0.0001 0.005 <0.0001	0.335 0.204 0.127	0.128
e^2^	Change in locomotion	Main effect of time Main effect of treatment Interaction	<0.0001 <0.0001 <0.0001	0.298 0.224 0.281	0.128

Experiment 1. The effect of estradiol on microglial, cytokine, and physiological response to LPS.

**Table 2:** Experiment 2 statistical analysis

	Dependent variable	Comparison	*p* value	Effect size	Power
f^1^	VMH mean Iba1-ir	Main effect of drug Main effect of treatment Interaction	0.0556 <0.0001	0.129 0.868	0.269
f^2^	VMH number of microglia	Main effect of drug Main effect of treatment Interaction	0.2079 <0.0001	0.060 0.794	0.261
f^3^	VMH total cell body area	Main effect of drug Main effect of treatment Interaction	0.4003 <0.0001	0.027 0.876	0.261
f^4^	VMH mean cell body area	Main effect of drug Main effect of treatment Interaction	0.513 <0.0001	0.017 0.927	0.261
f^5^	VMH total outgrowth	Main effect of drug Main effect of treatment Interaction	0.005 0.033	0.267 0.164	0.261
f^6^	VMH mean outgrowth	Main effect of drug Main effect of treatment Interaction	0.364 <0.0001	0.032 0.745	0.261
f^7^	VMH total processes	Main effect of drug Main effect of treatment Interaction	0.098 <0.0001	0.2101 0.775	0.261
f^8^	VMH total branches	Main effect of drug Main effect of treatment Interaction	0.011 <0.0001	0.224 0.570	0.261
f^9^	VMH microglial ratio	Main effect of ratio Main effect of treatment Interaction	<0.0001 <0.0001 <0.0001	0.773 0.546 0.440	0.184
g^1^	IL-1α 6 h	Main effect of drug Main effect of treatment Interaction	0.781 0.519 0.482	0.004 0.024 0.028	0.199
g^2^	IL-1β 6 h	Main effect of drug Main effect of treatment Interaction	0.347 <0.0001 0.01	0.052 0.767 0.332	0.191
g^3^	IL-6 6 h	Main effect of drug Main effect of treatment Interaction	0.040 0.001 0.040	0.213 0.566 0.213	0.199
g^4^	IL-10 6 h	Main effect of drug Main effect of treatment Interaction	0.682 <0.0001 0.642	0.011 0.737 0.013	0.183
g^5^	TNF-α 6 h	Main effect of drug Main effect of treatment Interaction	0.219 <0.0001 0.023	0.078 0.841 0.243	0.207
g^6^	Change in body weight 6 h	Main effect of drug Main effect of treatment Interaction	0.168 <0.0001 0.107	0.098 0.921 0.131	0.207
h^1^	IL-1α 24 h	Main effect of drug Main effect of treatment Interaction	0.325 0.041 0.946	0.057 0.223 0.000	0.191
h^2^	IL-1β 24 h	Two-way ANOVA	--	--	--
h^3^	IL-6 24 h	Main effect of drug Main effect of treatment Interaction	0.768 0.003 0.667	0.008 0.576 0.017	0.143
h^4^	IL-10 24 h	Main effect of drug Main effect of treatment Interaction	0.977 0.003 0.763	0.000 0.469 0.006	0.167
h^5^	TNF-α 24 h	Main effect of drug Main effect of treatment Interaction	0.441 0.180 0.178	0.035 0.103 0.104	0.191
h^6^	Change in body weight 24 h	Main effect of drug Main effect of treatment Interaction	0.281 <0.0001 0.752	0.058 0.784 0.005	0.214
i^1^	Change in temperature	Main effect of time Main effect of treatment Interaction	<0.0001 <0.0001 0.145	0.098 0.046 0.113	0.206
i^2^	Change in locomotion	Main effect of time Main effect of treatment Interaction	<0.0001 <0.0001 0.206	0.150 0.162 0.106	0.206

Experiment 2. The effect of formestane on the microglial, cytokine, and physiological response to LPS

### Experiment 1: does estradiol influence the microglial, cytokine, and physiological response to LPS?

**Figure 1.**
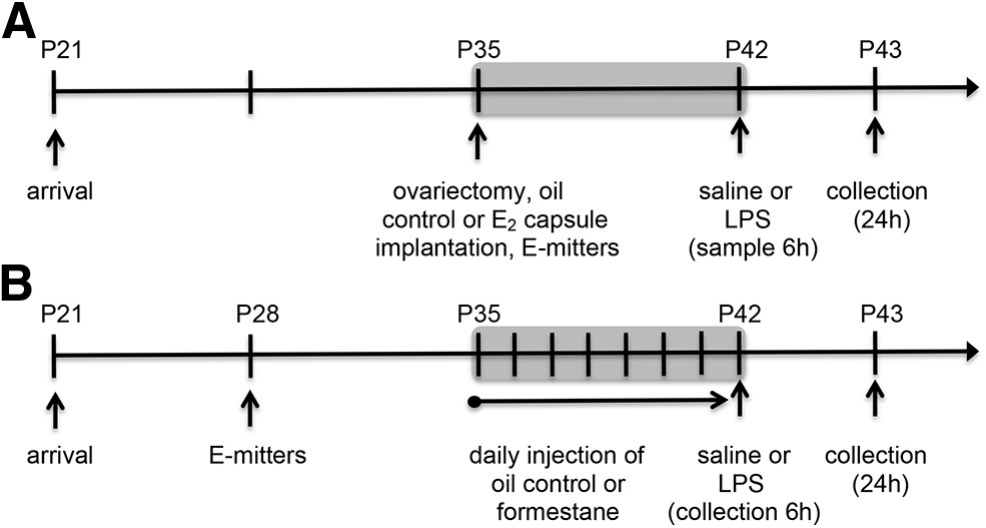
***A***, Experiment 1 timeline. ***B***, Experiment 2 timeline.

A total of 128 C57Bl/6 female mice were shipped on P21. Mice were ovariectomized and received a SILASTIC capsule implant on P35. Mice received an intraperitoneal injection of LPS or sterile saline vehicle on P42 (Fig. 1*A*). Thirty-two mice were euthanized 24 h following saline or LPS administration on P43, and brains were removed immediately and placed into 4% paraformaldehyde until processed for microglial analysis. Ninety-six mice were euthanized, decapitated, and trunk blood was collected either at 6 h postinjection (48 mice on P42) or 24 h postinjection (48 mice on P43) for cytokine analyses and physiological responses.

### Experiment 2: does formestane attenuate the microglial, cytokine, and physiological response to LPS?

A total of 128 C57Bl/6 female mice were shipped on P21. Mice received a daily injection of formestane or sesame oil for seven consecutive days from P35 to P42 (Fig. 1*B*). Mice received an intraperitoneal injection of LPS or sterile saline vehicle on P42. Twenty-four hours following saline or LPS administration (on P43), 32 mice were euthanized and brains were removed immediately and placed into 4% paraformaldehyde until processed for microglial analysis. Ninety-six mice were euthanized, decapitated, and trunk blood was collected either at 6 h postinjection (48 mice on P42) or 24 h postinjection (48 mice on P43) for cytokine analyses and physiological responses.

## Results

### Experiment 1

#### Estradiol increases basal microglial Iba1 immunoreactivity in the VMH

**Figure 2.**
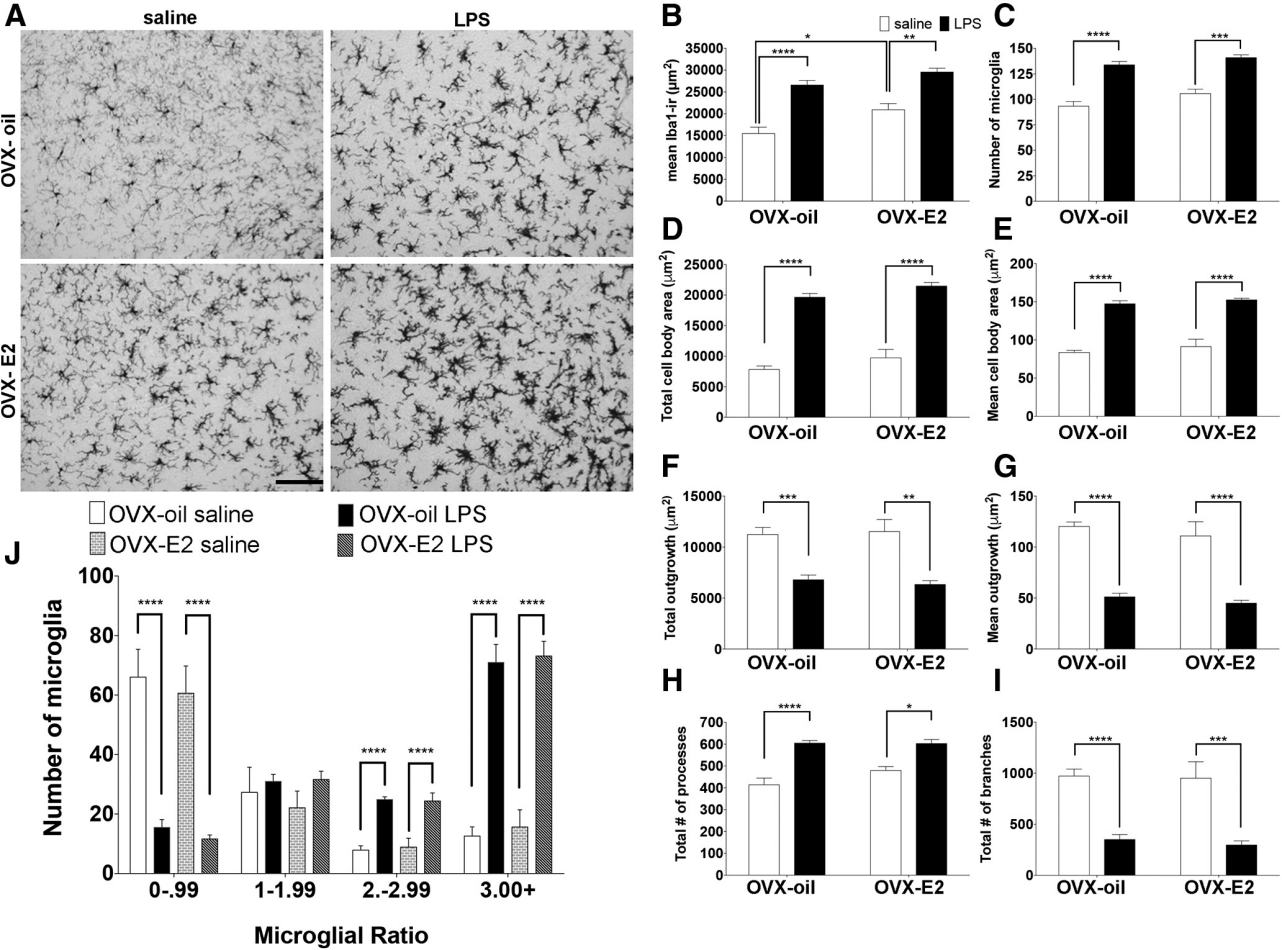
The effects of LPS on microglia in the VMH of ovariectomized (OVX) pubertal female mice that received an oil or estradiol (E_2_) capsule. ***A***, Representative photomicrographs of females treated with oil and saline, oil and LPS, E_2_ and saline, E_2_ and LPS. The effects of LPS on (***B***) microglial Iba1 immunoreactivity, (***C***) number of microglia, (***D***) total cell body area, (***E***) mean cell body area, (***F***) total outgrowth, (***G***) mean outgrowth, (***H***) total number of processes, and (***I***) mean number of branches of microglia. ***J***, Microglial heterogeneity. All data are represented as mean ± SEM with *p < 0.05, **p < 0.01, ***p < 0.001, ****p < 0.0001.

Estradiol increased basal microglial Iba1 immunoractivity^a1^ in mice treated with saline control. This increase in microglial Iba1 immunoreactivity is not due to differences in the total number of microglia^a2^ or differences in microglial cell body size^a3,a4^ (Fig. 2*C–E*). Overall, these results suggest that the estradiol-driven increase in the microglial Iba1 immunoreactivity might enhance immune activation in the VMH. As expected, microglia underwent robust changes in morphology following LPS treatment regardless of the hormone environment (Fig. 2). After LPS, there was an increase in microglia cell numbers^a2^ (Fig. 2*C*), increased soma size^a3,a4^ (Fig. 2*D*,*E*), and a decrease in their overall outgrowth^a5,a6^ (Fig. 2*F*,*G*), which was mainly driven by a decrease in microglial branches^a7,a8^ (Fig. 2*I*). The microglial ratio^a9^ analysis suggests that the cells undergoing these drastic morphologic changes are resident microglial cells within the VMH, as there is a robust decrease in the number of basal microglia (ratio 0–0.99) and a concomitant increase in macrophage-like microglia (ratio 3.00+; Fig. 2*J*).

#### LPS induces higher levels of plasma TNF-α in the presence of estradiol

**Figure 3.**
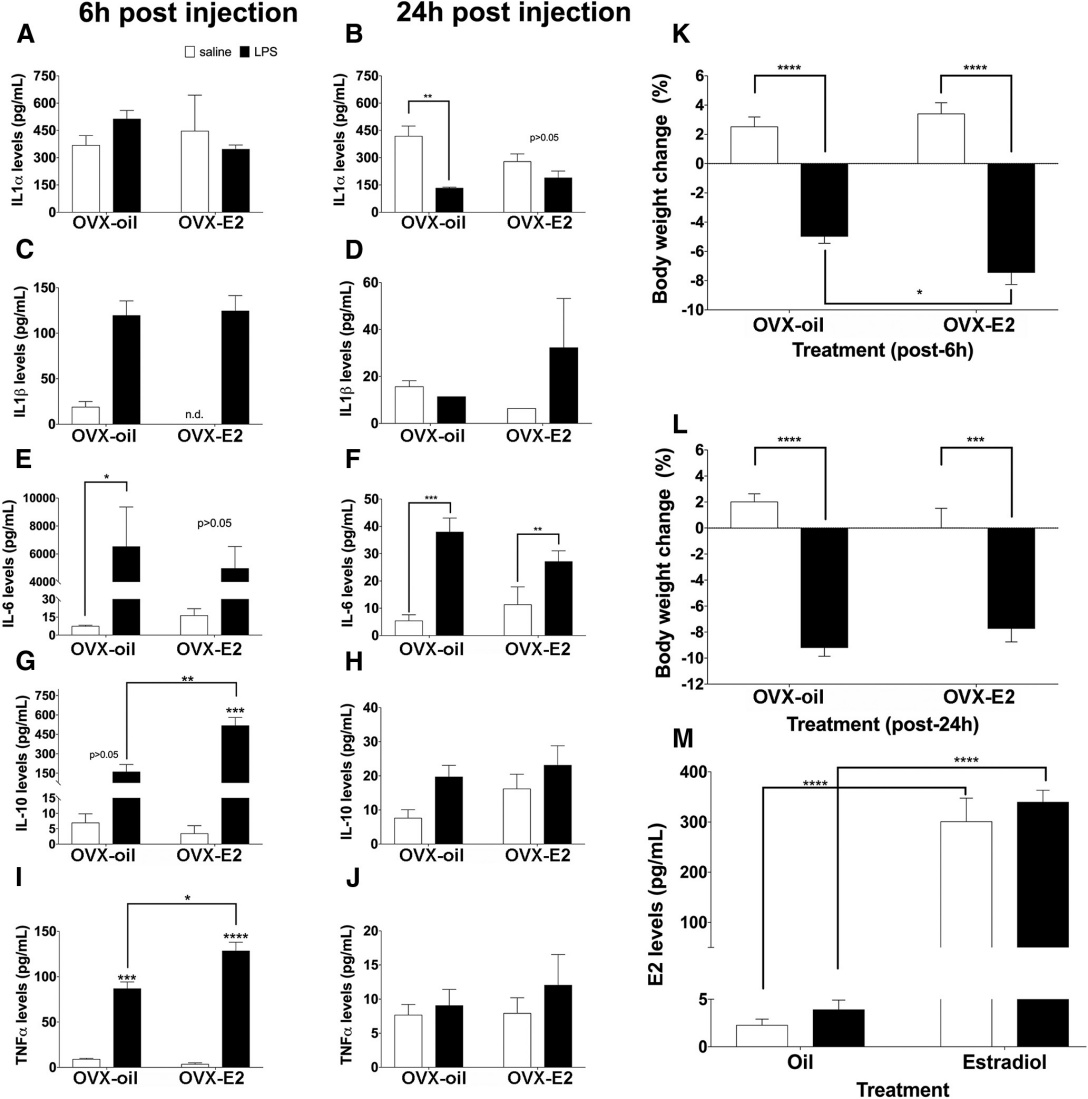
The effects of LPS on cytokine levels in ovariectomized (OVX) pubertal mice that received an oil or estradiol (E_2_) capsule. Levels of (***A***, ***B***) IL-1α, (***C***, ***D***) IL-1β, (***E***, ***F***) IL-6, (***G***, ***H***) IL-10, (***I***, ***J***) TNF-α at 6 h (***A***, ***C***, ***E***, ***G***, ***I***), or 24 h (***B***, ***D***, ***F***, ***H***, ***J***) postinjection. The effects of LPS on body weight collected at (***K***) 6 h or (***L***) 24 h postinjection. ***M***, Plasma E_2_ levels of OVX female mice implanted with an oil or E_2_ capsule. All data are represented as mean ± SEM with *p < 0.05, **p < 0.01, ***p < 0.001, ****p < 0.0001.

Plasma levels of IL-1α^b1,c1^, IL-1β^b2,c2^, IL-6^b3,c3^, IL-10^b4,c4^, and TNF-α^b5,c5^ were quantified at 6 and 24 h post-LPS administration. LPS caused a stronger induction of TNF-α and IL-10 in the presence of estradiol 6 h following LPS injection (Fig. 3*G*,*I*). IL-1α levels were similar between ovariectomized mice that received saline or LPS 6 h postinjection (Fig. 3*A*) but LPS administration caused a nearly three-fold decrease in IL-1α levels in ovariectomized, oil-controls 24 h following LPS administration (Fig. 3*B*). While LPS increased IL-6 levels in ovariectomized oil-controls and estradiol-treated mice 6 and 24 h post-LPS administration, the effect of LPS on IL-6 levels 6 h following LPS administration to ovariectomized, estradiol-treated mice did not reach statistical significance (Fig. 3*E*,*F*). LPS caused a decrease in body weight 6h^b6^ and 24h^c6^ after administration, compared with saline-treated controls (Fig. 3*K*,*L*). The body weight change confirms that the LPS treatment reduced food intake (Fig. 3*K*,*L*). Finally, the estradiol levels^d1^ further confirmed the appropriate delivery and release of estradiol from the capsules (Fig. 3*M*).

#### LPS causes hypothermia in the presence of estradiol

**Figure 4.**
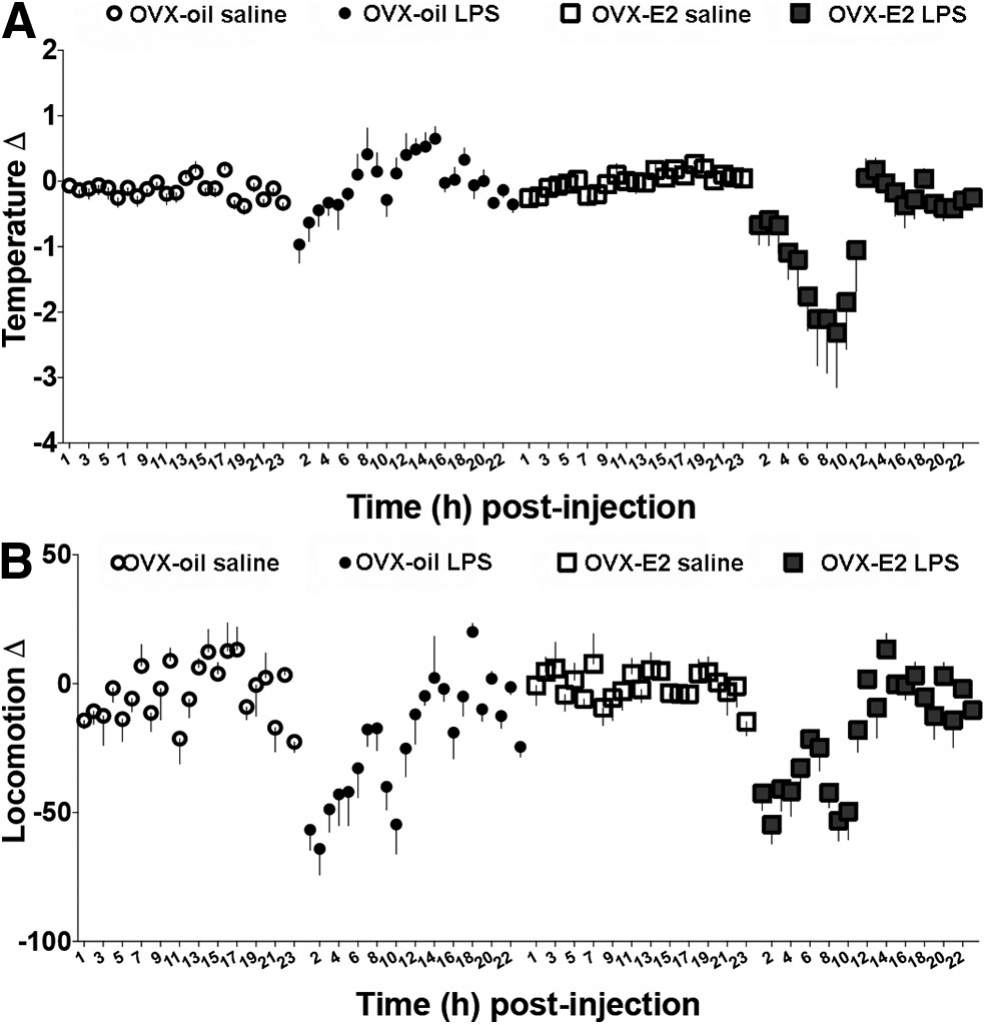
The effects of LPS administration on (***A***) temperature and (***B***) locomotion in ovariectomized (OVX) pubertal females that received an oil or estradiol (E_2_) capsule (mean ± SEM).

LPS caused hypothermia *only* in the presence of estradiol^e1^ (Fig. 4*A*). This temperature change is not attributable to changes in locomotor activity, because estradiol-treated and oil-treated control mice showed a similar reduction in locomotion^e2^ following LPS administration (Fig. 4*A*,*B*). Additionally, estradiol-treated and oil-treated controls returned to baseline locomotor activity at a similar time point (Fig. 4*B*).

### Experiment 2

#### Formestane decreases basal microglial branching complexity in the VMH

**Figure 5.**
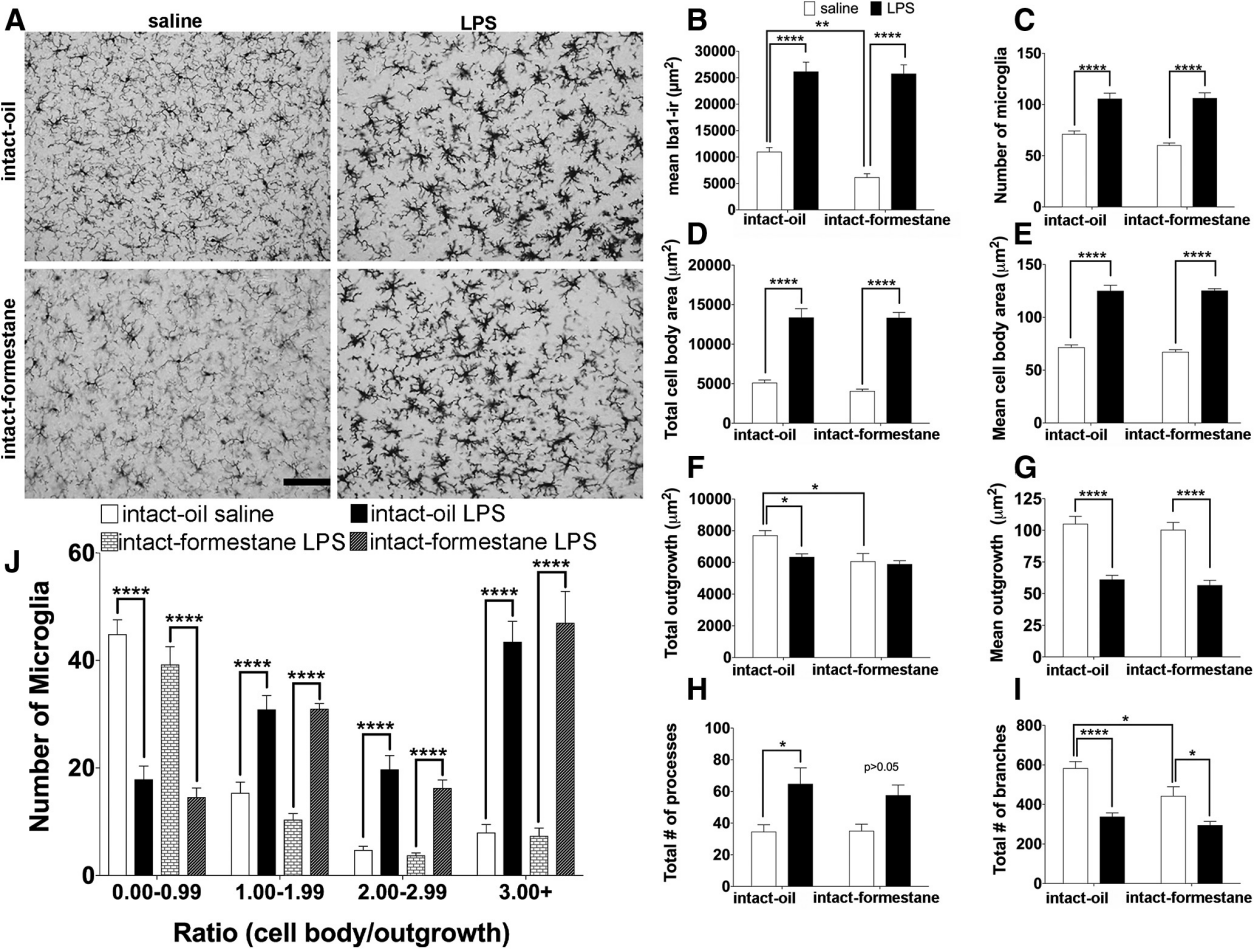
The effects of LPS on microglia in the VMH of intact pubertal female mice that received injections of either oil or the aromatase inhibitor formestane. ***A***, Representative photomicrographs of females treated with oil and saline, oil and LPS, formestane and saline, formestane and LPS. The effects of LPS on (***B***) microglial Iba1 immunoreactivity, (***C***) number of microglia, (***D***) total cell body area, (***E***) mean cell body area, (***F***) total outgrowth, (***G***) mean outgrowth, (***H***) total number of processes, and (***I***) mean number of branches of microglia. ***J***, Microglial heterogeneity. All data are represented as mean ± SEM with *p < 0.05, **p < 0.01, ***p < 0.001, ****p < 0.0001.

The aromatase inhibitor, formestane, prevents the synthesis of estradiol. Ovary-intact mice treated with oil show higher levels of microglial Iba1 immunoreactivity at basal levels compared with mice treated with formestane^f1^ (Fig. 5*B*). The effect of formestane seems to be mediated via effects on microglial outgrowth^f5,f6^ (Fig. 5*F*,*I*) but not by the number of microglia^e2^ (Fig. 5*C*) or by the cell body size^f3,f4^ (Fig. 5*D*,*E*). Specifically, the administration of formestane reduces the complexity of microglial branching^f8^ (Fig. 5*I*). As expected, microglia underwent robust changes in morphology following LPS treatment regardless of the hormone environment (Fig. 5). After LPS treatment, there was an increase in microglia cell numbers (Fig. 5*C*), increased soma size (Fig. 5*D*,*E*), and a decrease in their overall outgrowth (Fig. 5*F*,*G*), which was mainly driven by a decrease in microglial branches (Fig. 5*I*). The microglial ratio analysis^f9^ suggests that the majority of microglia undergoing these morphologic changes are resident microglial cells within the VMH, as there is a robust decrease in the number of basal microglia (ratio 0–0.99) and a concomitant increase in macrophage-like microglia (ratio 3.00+; Fig. 5*J*).

#### Formestane attenuates the induction of plasma IL-6 and IL-1β following LPS

**Figure 6.**
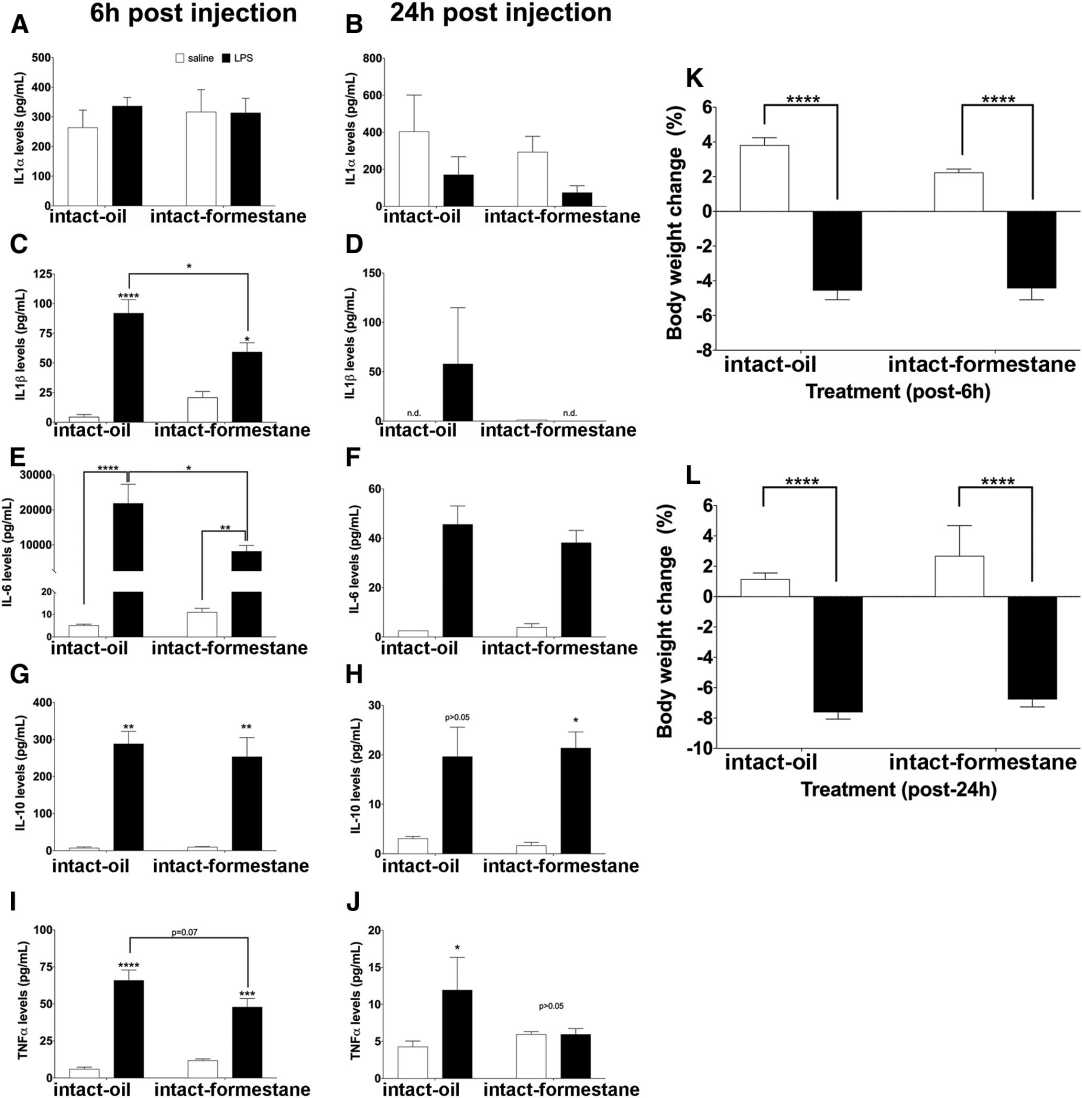
The effects of LPS on cytokine levels in intact pubertal mice that received injections of either oil or the aromatase inhibitor formestane. Levels of (***A***, ***B***) IL-1α, (***C***, ***D***) IL-1β, (***E***, ***F***) IL-6, (***G***, ***H***) IL-10, (***I***, ***J***) TNF-α at 6 h (***A***, ***C***, ***E***, ***G***, ***I***) or 24 h (***B***, ***D***, ***F***, ***H***, ***J***) postinjection. The effects of LPS on body weight collected at (***K***) 6 h or (***L***) 24 h postinjection. All data are represented as mean ± SEM with *p < 0.05, **p < 0.01, ***p < 0.001, ****p < 0.0001.

Plasma levels of IL-1α^g1,h1^, IL-1β^g2,h2^, IL-6^g3,h3^, IL-10^g4,h4^, and TNF-α^g5,h5^ were quantified at 6 and 24 h post-LPS administration. In ovary-intact oil controls, LPS causes a stronger induction of IL-1β and IL-6, while formestane attenuates it 6 h following LPS administration (Fig. 6*C*,*E*). Not surprisingly, LPS increased the levels of IL-10 and TNF-α, but the hormone environment did not seem to modulate the levels of these cytokines (Fig. 6*G*). Overall, the levels of almost all of these cytokines had returned to baseline 24 h following LPS administration, suggesting that they are temporally regulated. Lastly, the body weight change^g6,h6^ suggests that the LPS treatment reduced food intake and/or induced changes in metabolism, both of which are consistent with sickness behavior (Fig. 6*K*,*L*). The estradiol levels for both groups were below the level of detection (3 pg/ml) for this experiment (data not shown).

#### Formestane blocks LPS-induced hypothermia seen in ovary-intact controls

**Figure 7.**
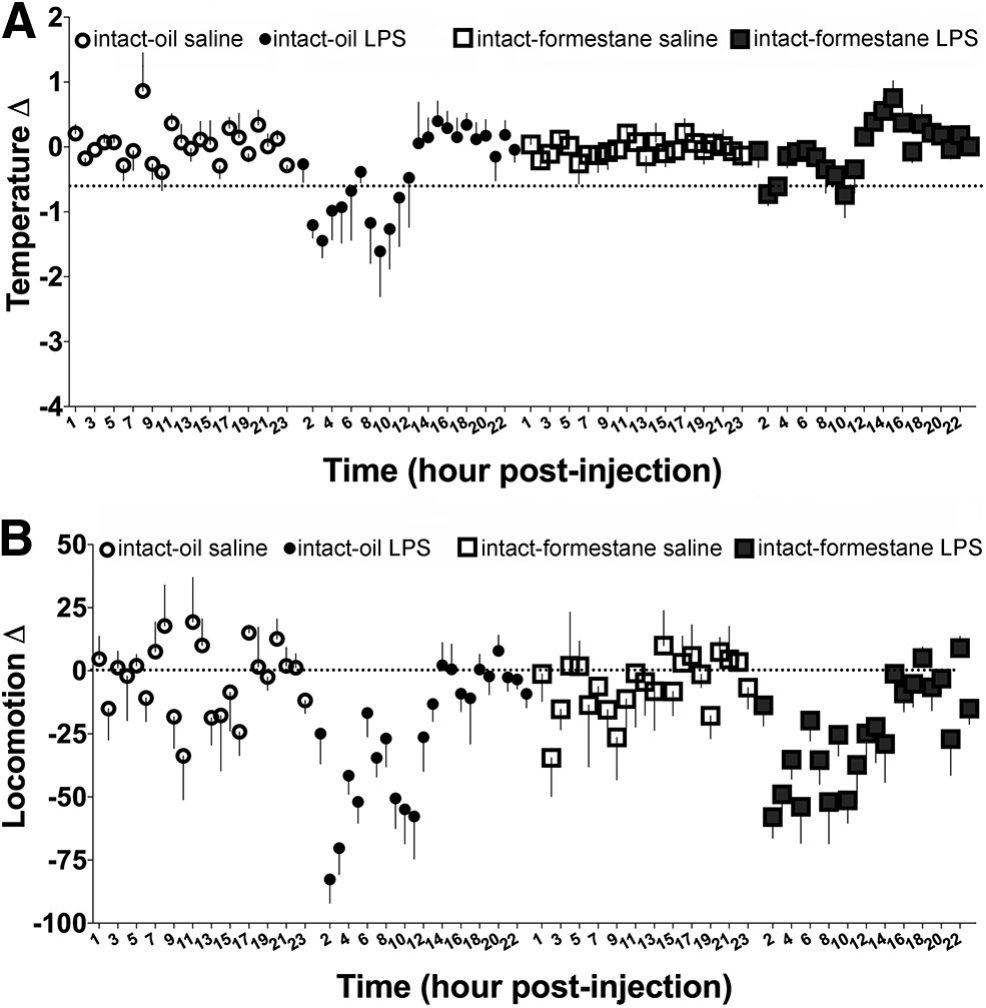
The effects of LPS administration on (***A***) temperature and (***B***) locomotion in intact pubertal females that received injections of either oil or the aromatase inhibitor formestane (mean ± SEM).

LPS caused hypothermia only in the presence of estradiol^i1^ (Fig. 7*A*). Only ovary-intact control mice showed a hypothermic response after LPS administration, while formestane blocked this induction (Fig. 7*A*). This temperature change cannot be attributed to changes in locomotor activity^i2^, because LPS-treated mice showed a similar reduction in locomotion following LPS administration regardless of whether the mice were able to synthesize estradiol (Fig. 7*B*).

## Discussion

Previous research has demonstrated that female mice are vulnerable to LPS during the sensitive period of pubertal development. First, LPS exposure during the peripubertal period, but not in adulthood, alters multiple behaviors mediated by steroid hormones in adulthood (Laroche et al., 2009a,b; Ismail et al., 2011; Olesen et al., 2011). Second, the majority of microglia in the VMH of gonadally intact females are characterized by longer and thicker processes during the peripubertal period in contrast to the thinly ramified microglial phenotype observed in adulthood, and the microglia in the VMH of pubertal mice have a stronger response to LPS, compared with microglia of adult mice receiving the same treatment (Holder and Blaustein, 2017). Third, there is a reduction in the concentration of ERα in the VMH if mice experience a shipping stressor during the peripubertal sensitive period (Ismail et al., 2011). Together, these data suggest that ovarian hormones may be important in conferring vulnerability to LPS during the peripubertal period. Here, we tested the hypothesis that estradiol confers vulnerability to LPS during the peripubertal period by modulating microglia. The present work indicates that estradiol increases microglial Iba1 immunoreactivity and formestane treatment alters microglial branching complexity in the VMH and amplifies the LPS-induced immune response in female mice during pubertal development. The findings suggest that estradiol enhances immune activation during the peripubertal sensitive period and may confer vulnerability to LPS.

Estradiol elevates basal microglial Iba1 immunoreactivity and formestane administration reduces microglial branching in the VMH. The microglial alterations may enhance the ability of microglia to respond more rapidly to perturbations in the microenvironment. Alternatively, it is possible that this type of microglia may act as the main driver of phagocytic activity during sensitive periods. While amoeboid microglia are typically associated with macrophage functions, new evidence shows that ramified microglia in the developing cerebellum contain phagocytic cups that are associated with cleaved caspase-3 expression and cell nuclei fragmentation in a brain-region and time-dependent manner (Perez-Pouchoulen et al., 2015). Future studies should test whether these microglial ramifications contain phagocytic cups in estradiol treated mice. This may be one mechanism that could underlie the reduction in ER-positive cells in the VMH of mice exposed to a stressor during peripubertal development. In this study we did not investigate estrogen-receptor expression in microglia. It is possible that estrogen receptors are expressed only in a subset of microglia and we might be overlooking subtle nuances in microglial morphology that could inform microglial function. Future experiments should focus on studying ER-expressing microglia in the VMH to evaluate the effects of estradiol on microglial function in that population.

The increase in microglial Iba1 immunoreactivity seen in the VMH of estradiol-treated mice is brain-region specific. Estradiol did not affect the microglial outgrowth in the AVPv or the ARC, areas which also express high levels of estrogen-receptors and undergo remodeling during the peripubertal period (data not shown). This region specificity may arise if the VMH is able to integrate signals from immune activity with the estradiol activity. For example, microinjections of prostaglandin E_2_ (PGE_2_) into the VMH causes a febrile response in rabbits (Morimoto et al., 1988). In addition, estradiol induces PGE_2_ synthesis and regulates microglial morphology and activity in the preoptic area in neonatal male pups (Amateau and McCarthy, 2004; Lenz et al., 2013). As LPS is able to stimulate the production of PGE_2_ (for review, see Ricciotti and FitzGerald, 2011), additional experiments should investigate whether a similar interaction between estradiol and PGE_2_ occurs in the VMH of pubertal female mice.

The notion that estradiol increases microglial Iba1 immunoreactivity, which can be associated with an enhanced response to immune activation, during pubertal development is supported by the cytokine, thermoregulatory, and metabolic changes observed in mice. LPS induces higher levels of TNF-α, hypothermia, and greater weight loss only in the presence of estradiol. Fever is a typical response to infection, but hypothermia is only induced in severe cases of inflammation (Romanovsky et al., 1996). Overall, these data show that these physiological changes are temporally regulated, and they suggest that estradiol is amplifying the LPS-induced immune response.

There are a couple of important caveats regarding the contributions of estradiol and other ovarian hormones to keep in mind when interpreting these data. First, these data indicate that presence of estradiol is required for the increased vulnerability to LPS during the peripubertal period; however, estradiol may not be the sole driver. In fact, one reason for the differences in microglial branching and cytokine expression between experiments 1 and 2 may be due to these other factors. Formestane, as an aromatase inhibitor, blocks the synthesis of estrogens, whereas the removal of the ovaries would also impact the estrogens and other ovarian hormones such as progestins. Therefore, adding back estradiol after removal of the ovaries is not the opposite of blocking synthesis of estrogens all over the body. The second relates to the estradiol levels. Although the route of administration of estradiol used in this study rescues the effects of ovariectomy on several markers of pubertal maturation (Clarkson et al., 2009), it is unknown whether the concentration of estradiol is within the physiological range for peripubertal mice. Therefore, we cannot exclude the possibility that the level of estradiol administered to peripubertal animals in experiment 1 was supraphysiological. Our estradiol assay was insufficiently sensitive to measure the blood levels of estradiol in the ovary-intact mice in experiment 2. The low levels of estradiol may be due to the developmental status of the mice, as the levels of estradiol increase over the course of pubertal development (deCatanzaro et al., 2004). The dose and injection procedure of formestane reduces brain levels of estradiol and estradiol-dependent changes in dendritic morphology in perinatal rats (Amateau et al., 2004; Dean et al., 2012); thus, the possibility remains that future experiments would show differences in the brain levels of estradiol in pubertal female mice treated formestane. However, the efficacy of formestane administration on the inhibition of aromatization and estradiol levels in the current study could not be determined.

Another caveat to keep in mind when interpreting these microglia data are that they reflect the interaction between the hormone environment and an immune challenge at a single time point (24 h postinjection). Our behavioral, physiological, cytokine, and metabolic data showed dynamic changes in the immune response within the first 24 h after LPS administration. It is possible that the effect of estradiol on microglial morphology is also temporally regulated. In addition, the cytokine analysis was performed using plasma samples, so the cytokine levels in the hypothalamus are unknown. Therefore, future studies should be conducted to determine the cytokine levels in hypothalamic homogenates. Lastly, it is important to investigate whether these manipulations that impact the microglia also prevent the altered hormone-responsive behaviors in adulthood.

While both, pubertal males and females, are sensitive to shipping from the breeder facility as indicated by reduction in the sexual behaviors in adulthood, females show a more robust response (Laroche et al., 2009b). It is unknown whether this is because there is a sex differences in the vulnerability or whether the timing chosen for the stressor is not ideal in male mice. The pattern of events that lead to sexual differentiation differs greatly between the sexes, so there may not be an objective way to determine the most comparable time to expose pubertal males to these stressors that would provide meaningful sex difference information. Therefore, although this work supports a robust effect of certain stressors during the peripubertal period in females, it does not directly address sex as a biological variable.

The goal of the present work was to examine the potential cellular underpinnings of the vulnerability of female mice to LPS administered in the peripubertal period. The present work suggests that estradiol may be a key factor in conferring vulnerability to LPS during the peripubertal sensitive period. While not tested directly, the increase in estradiol-driven microglial Iba1 immunoreactivity may suggest that microglia in the VMH are more active during the peripubertal period. Regardless, estradiol seems to amplify the LPS-induced responses in female mice during pubertal development at the microglial, cytokine, and thermoregulatory levels. It is possible that there is an enhanced interaction between the endocrine and immune systems as part of typical pubertal development. However, exposure to an immune stressor like LPS intensifies the immune signaling pathways to a threshold that may have detrimental effects. Although the present experiments do not directly address the issue of the effects of peripubertal immune challenge or shipping stress on later behavioral response to estradiol, they suggest that estradiol enhances the effect of LPS during the pubertal sensitive period.

## Synthesis

Reviewing Editor: Margaret McCarthy, Univ of Maryland School of Medicine Decisions are customarily a result of the Reviewing Editor and the peer reviewers coming together and discussing their recommendations until a consensus is reached. When revisions are invited, a fact-based synthesis statement explaining their decision and outlining what is needed to prepare a revision will be listed below. The following reviewer(s) agreed to reveal their identity: Colin Saldanha.

You are fortunate to have 2 expert reviewers who provide a number of important suggestions for improvement of this manuscript. Since each of them note distinct but useful suggestions I have provided them below in unedited form. Please give thoughtful consideration to each comment and use them as guidance for additional modifications if warranted in regards to precision in your wording and care in your presentation.

Reviewer #1

This paper builds on the observation that stress (shipping) and/or activation of the innate immune system via an injection of LPS during the peripubertal period, can disrupt adult sex behavior in mice. The hypothesis being tested in this set of experiments is that ovarian estrogens (estradiol, E2) may modulate microglial reactivity in the VMH, thereby affecting cytokine levels and sickness associated behaviors in peripubertal mice. The researchers test OVX females with or without E2 replacement and measure basal Iba-1 (microglial) activity and Iba-1 expression, plasma cytokine and sickness behaviors following a single injection of LPS. In another experiment aromatase activity if chronically inhibited (with oil controls), basal Iba-1 expression, and Iba-1 expression, cytokine and sickness behaviors following a single injection of LPS were measured.

The authors show that E2 replacement and aromatase inhibition increase or decrease basal microglial activity respectively. Further, LPS-induced cytokine expression and some sickness behaviors are exaggerated or mitigated in E2 replaced or formestane-treated mice respectively. They conclude that endogenous estrogens may confer a susceptibility to LPS in peripubertal mice via actions on the reactivity of microglia in the VMH. The paper is generally well written (but see below), and the data are exhaustively described. The authors are to be commended for the quality of the ICC and associated images which clearly show an increase in the intensity of basal and LPS-induced microglial reactivity following E2 replacement and a decrease in the same measures following formestane.

1. There is no validation of the efficacy of formestane in experiment 2. The authors show data for circulating E2 levels in the first experiment (E2 replacement), but fail to show any measures of aromatase inhibition in experiment 2. Were these data not collected? Given the plasma was drawn, it would be very important to show that formestane at the dose and duration administered, was sufficient to lower endogenous E2 levels. In the absence of plasma data, perhaps the authors could show some indirect (bioassay) measures that reflect circulating E2. The Amateau et al, 2004 reference is used to suggest that the dose used lowers circulating E2, but I believe the paper cited measured neural E2 levels early in development (Editors note: I can confirm this is the case). The differences between perinatal and juvenile rodents is considerable. The authors need to justify the dose of formestane used and suggest some way in which the reader can appreciate the efficacy of the dose and mode of administration used at this stage of development.

2. While the exhaustive description of the measures and statistics in Tables 1 and 2 is appreciated, a bit of re-organization may help the reader sift through them better. I suggest listing all the 6h measures (plasma and behavior) before all the 24hr data. Additionally, main effects are typically described before interactions, and listing them as such in column 6 would be helpful. Finally, columns 3 and 4 (data type and statistical test) could be removed and described in a few sentences in the text.

3. The generalizability of these findings is somewhat limited given that there is no comparison to other ages outside the peripubertal period. I recognize the amount of work the authors of have done here, but given that the anti-inflammatory effects of estrogens are very well described in songbirds and adult rodents, the significance of these observations becomes all the more intriguing. Do the authors believe that the susceptibility conferred by estrogens in the VMH occurs only during the peripubertal period? Do these effects generalize to other ages and/or brain areas?

4. The authors state the following, “Three bilateral photomicrographs of carefully matched sections of ventromedial nucleus of the hypothalamus (VMH) were taken from each animal...”. What criteria were used to achieve this matching? Landmarks? Anterior/posterior plane relative to Bregma? The authors need to provide some justification for the matching.

Reviewer #2

This manuscript details nice mechanistic experiments showing that the presence of estradiol is required for the increased vulnerability of females to LPS during the sensitive peripubertal period. However, there are multiple issues with terminology, interpretation, and caveats of the methodology that prevent it from being suitable for publication in its current form. I have listed my comments and suggestions in the order they appear below:

• Abstract: “estradiol elevates basal microglial density by increasing the number of microglial branches in the VMH”. What does this mean? Microglial density implies that the number of cell bodies of microglia per unit area is increased (not the branching complexity).

• Methods, page 7: Mice were not perfused prior to collecting brains, so the Iba1 IHC is going to stain not just microglia, but monocytes and macrophages in and around the blood vessels. This should be mentioned as a caveat in the methods and/or discussion.

• Methods, page 10: Why were cytokines analyzed in plasma only, and not in the actual brain region where you analyzed microglia? The immune response in the actual brain (measured via an ELISA of the hypothalamus homogenate) would have been informative (and may be different from the peripheral cytokine response), so perhaps you should mention this as a future direction in the discussion.

• Methods, page 11 (and same on 12): “For microglial analysis, a separate group of thirty-two mice was euthanized, and brains were removed from the skull 24h later (on P43).” This sentence is worded confusingly-do you mean that the mice were euthanized, then they sat around for 24 h until the brains were removed? This is what it sounds like based on how you’ve written it, but experimentally doesn't make much sense. Were these mice also injected with LPS or saline and euthanized 24 h later?

• Results, page 12: “Estradiol increased basal microglial density in mice treated with saline control. This estradiol-driven rise in microglial reactivity is due to increased microglial outgrowth, which refers to microglial processes and branches.” Again, the “density” term should be changed to “branching complexity” or something along these lines if you are referring to changes in the processes. I would also recommend not using the term “reactivity” here, because more branching complexity is traditionally associated with less reactive/amoeboid microglia, so the way you have used it here is confusing.

• Figure 3M: The black vs. white no longer mean saline vs. LPS, which is confusing. Could you reverse the x-axis and legend so as to maintain consistency with the rest of the figures?

• Figure 5J: legends appear to be labeled incorrectly for “intact-formestane LPS", it appears twice- the first time it should be “intact-formestane saline”.

• Figure 6: You are missing the 6h and 24h labels over each column this time. Also, the cytokines changed after formestane are not the same as the ones changed after estrogen, suggesting that the mechanism may not be quite overlapping. This should be mentioned in the discussion as a possible caveat. Why did you not measure estrogen levels in the intact mice? This would be helpful to include so we know if the levels you induced in the 1st experiment are relevant to natural levels at this age, and also to show that the formestane treatment worked.

• Discussion, page 16: “The present work indicates that estradiol increases immune surveillance in the VMH...” This statement is far too over-reaching based on what measurements were made in the current paper. The authors did not measure microglial process motility, which would be more indicative of immune surveillance. The subtle changes in microglial morphology due to estradiol do not link directly to functional changes in immune surveillance (and there is not good evidence of what microglial morphology means in terms of function, especially during developmental periods). Thus, please temper your statements to reflect what you actually measured.

• Discussion, page 16: “First, estradiol elevates basal microglial density by increasing the number of microglial branches in the VMH.” As elsewhere, please do not use the term ‘microglial density' for changes in process branching.

• Discussion, page 17: “Importantly, the higher levels of microglial outgrowth seen in the VMH of estradiol-treated mice is brain-region specific.” More discussion of why the authors think the changes are specific to the VMH would be useful to the reader.

• Discussion, page 18: “Although the present experiments do not directly address the issue of the effects of peripubertal immune challenge or shipping stress on later behavioral response to estradiol, they suggest a potential mechanism underlying the biological underpinnings of this long-term change in adult behavior.” I’m not sure I agree that these studies suggest a potential mechanism underlying the long-term change in behavior. You have only looked at the acute immune and microglial response to LPS (which is not really a stressor, but an immune challenge, and should be worded as such), so you have no idea from these data what mechanisms may underlie long-term changes in behavior (e.g., epigenetic changes, circuit rewiring, etc.). Therefore, the wording of your discussion needs to be tempered and adjusted to reflect your findings. In the abstract and significance statement, you make somewhat similar claims that “these data suggest one mechanism by which estradiol confers vulnerability to LPS during the pubertal sensitive period.” Again, this is not really true- you have just shown that the presence of estradiol confers vulnerability to LPS during the pubertal sensitive period, but you haven't explored further molecular mechanisms underlying this vulnerability. Please modify and be more specific with your claims here as well.

• Discussion, general: Your discussion (and introduction) should also include mention of whether males have been previously shown to be sensitive to LPS or stressors during the peripubertal period, and whether testosterone (and/or estradiol) have been implicated there as well, in order to fully address sex as a biological variable.
